# Remaining Useful Life Prediction for Rolling Bearings Based on TCN–Transformer Networks Using Vibration Signals

**DOI:** 10.3390/s25113571

**Published:** 2025-06-05

**Authors:** Xiaochao Jin, Yaping Ji, Shiteng Li, Kailang Lv, Jianzheng Xu, Haonan Jiang, Shengnan Fu

**Affiliations:** 1 Xi’an Key Laboratory of Extreme Environment and Protection Technology, School of Aerospace Engineering, Xi’an Jiaotong University, Xi’an 710049, China; jinxiaochao@xjtu.edu.cn (X.J.);; 2 Xi’an Institute of Electromechanical Information Technology, Xi’an 710065, China; 3 Xi’an Modern Control Technology Research Institute, Xi’an 710065, China

**Keywords:** rolling bearings, deep learning, remaining useful life prediction, health index, performance degradation

## Abstract

Remaining useful life (RUL) prediction plays a core role in industrial prognostics and health management (PHM), requiring data-driven models with higher predictive capability for accurate long time series prediction. Developing reliable deep learning-based models based on multi-sensor monitoring data is fundamental for accurately predicting vibration trends during bearing operation and is crucial for bearing fault diagnosis and RUL prediction. In this work, a method for constructing a health index based on vibration signal is developed to describe the performance features of rolling bearings, which mainly includes feature extraction, sensitive feature index selection, dimensionality reduction, and normalization methods. In addition, a new RUL prediction method, TCN–Transformer, is developed which can efficiently learn and integrate local and global features of vibration signals, addressing the long time series prediction problem in RUL prediction. The TCN extracts local features, while the Transformer learns global features, both of which are seamlessly integrated through a specially designed feature fusion attention module. Both the health indicator (HI) constructed from extracted time domain and frequency domain feature parameters and the RUL prediction method were rigorously validated using the IEEE PHM 2012 Data Challenge dataset for rolling bearing prognostics. By employing the proposed HI construction method, the average comprehensive bearing performance index, used to evaluate RUL prediction accuracy, is improved by 8.69% across the entire dataset compared to the original feature-based composite index. The proposed RUL prediction model can more accurately predict the RUL of rolling bearings under different conditions, reducing the RMSE and MAE by 14.62% and 9.26%, respectively, and improving the SCORE by 13.04%. These results underscore the efficacy and superiority of our approach in RUL prediction of rotating machinery across varying conditions.

## 1. Introduction

The deep learning method has demonstrated exceptional performance across various aspects of the industrial sector, particularly in health monitoring and the intelligent operation and maintenance of critical industrial components. Prognostics and health management (PHM), which includes fault detection, diagnosis, and remaining useful life (RUL) prediction, has recently attracted great research interest [1,2,3,4]. PHM based on using deep learning methods has great potential in the capability of deploying these maintenance strategies provides the opportunity of setting efficient, just-in-time and just-right maintenance strategies [5,6]. Rolling bearings are key components in rotating machinery, directly affecting the safety of the entire mechanical system. Vibration monitoring is crucial for early fault detection, localization, and differentiation [7,8,9,10,11]. Developing reliable deep learning-based models based on multi-sensor monitoring data is fundamental for accurately predicting vibration trends during bearing operation and is crucial for bearing fault diagnosis and RUL prediction [12,13,14,15].

Generally, PHM primarily employs various techniques to analyze monitoring data, extract discriminative knowledge, and assess the health status of mechanical equipment. It is generally expected to achieve three functions: health status monitoring, fault diagnosis, and RUL prediction. Among them, RUL estimation is considered to be the most challenging task because the continuous use time of mechanical equipment is inconsistent, and it is difficult to accurately extract sensitive degradation features under different degradation modes [16,17,18]. Constructing a health index (HI) to describe performance features from continuous operational signals is a critical prerequisite for effective RUL prediction using data-driven methods. Deep learning-based methods, such as Recurrent Neural Networks (RNNs) and their variants, are increasingly being utilized to extract identifiable degradation features through their specialized cyclic memory structures, establishing themselves as a prominent area of research [19].

Current RUL prediction is generally divided into three categories: physical model-based methods, data-driven techniques, and hybrid strategies [20]. Physical model-based methods are established according to component damage mechanisms and deterioration laws of specific failure modes, with prominent examples including Fatigue Crack Growth (FCG) [21] and Fatigue Spall Progression Life (FSPL) models [22]. These approaches describe structural degradation evolution through physical mechanisms but typically need substantial prior knowledge, making accurate degradation estimation difficult in complex conditions [23,24,25]. In contrast, data-driven methods construct models based on sensor data without depending on particular degradation patterns, using extensive historical data for empirical learning [26]. With the progress of machine learning, data-driven approaches are being applied more frequently in industrial applications to learn empirical patterns from historical data.

The advent of machine learning technologies has significantly influenced RUL prediction development. Bearing sensor monitoring data are time series data. Therefore, the problem of bearing degradation trend prediction is essentially a regression problem related to time series [27]. Consequently, the ability of the constructed model to learn effective time information is crucial to the final prediction result. Traditional machine learning-based prediction methods usually require a feature extraction process before prediction. The procedure involves first extracting a set of features from condition monitoring data and then inputting these features into a machine learning model to perform the RUL prediction task. Traditional machine learning-based methods do not consider the correlations between time series signals that reflect the changes in the health state of mechanical equipment. Additionally, they typically rely on manually extracting features from raw sensor data, estimating health indicators, degradation states, and predicting RUL using failure thresholds. With the advancements in deep learning technologies, such as Convolutional Neural Networks (CNNs), Recurrent Neural Networks (RNNs), and Transformers [28], the application of this method has garnered increasing attention. Deep learning techniques possess the capability to analyze high-dimensional data and automatically extract features. Deep learning-based methods have emerged and achieved remarkable results across various fields, primarily due to their robust capability to map the relationship between degradation paths and measurement data, and their ability to automatically learn degradation features, thus eliminating the need for manual feature extraction and expert knowledge of mechanical systems. Among these methods, the RNN and Long Short-Term Memory (LSTM) models have been particularly prominent in RUL prediction tasks, effectively utilizing temporal information [29]. Additionally, both BiLSTM and LSTM models demonstrate strong performance in time domain health monitoring applications [30,31].

However, as the service life of mechanical equipment continues to extend, long-term degradation behavior prediction becomes increasingly essential, and the shortcomings of RNN-based prediction frameworks are gradually exposed, mainly in the following aspects: (1) RNNs’ inability to process time series in parallel, necessitating strict chronological order; (2) difficulties in memorizing long-term historical data, leading to error accumulation in predictions; and (3) increased computational complexity due to the intricate gating structures of RNN variants like LSTM and Gated Recurrent Units (GRUs) [32]. Therefore, how to process long time series efficiently and accurately has become an urgent problem that needs to be solved.

Furthermore, those existing models have fully succeeded in effectively learning local correlation features and global features, which is the key to RUL prediction. Recently, Transformer-based models have successfully learned global features for different types of data, including time series [33]. Their attention mechanisms enhance training speed, support parallel computing, and improve accuracy compared to RNNs. The unique output mechanism of the Transformer-based models can greatly reduce the error accumulation in the prediction process. In response to the unique challenges in time series modeling tasks, many variants of the Transformer model have been developed, which have been successfully applied to a variety of time series tasks, including but not limited to prediction, anomaly identification, and classification problems. However, directly applying these models to multivariate time series data for bearing vibration prediction may not fully utilize the inherent characteristics of the data, such as temporal dynamics and the relationship between different dimensions. It often fails to capture the overall feature distribution of the time series, which limits its prediction effect.

In contrast, CNN-based architectures demonstrate superior local pattern extraction capabilities through their hierarchical filter structures [34]. Temporal Convolutional Networks (TCNs) [35] further enhance this advantage by incorporating dilated convolutional layers, preserving CNN’s inherent local feature extraction while effectively capturing long-range temporal dependencies in sequential data [36]. This complementary functionality motivates the integration of Transformer architectures with TCNs for enhanced time series representation learning. While existing studies have employed serial arrangements of these models for temporal data processing [37], such sequential architectures often neglect the intrinsic interplay between local and global features in vibration signals. A more effective approach requires independent learning of hierarchical features, followed by deliberate fusion through optimized integration mechanisms.

The main contributions of this study are summarized as follows:

A method for constructing a health index based on vibration signal (HIVS) is developed to describe the performance features of rolling bearings. The bearing vibration signals can be decomposed into eight wave packets using wavelet transformation, resulting in an initial feature set comprising 32 feature indexes that capture the signal characteristics. These indexes are derived from the original vibration signals across the time domain, frequency domain, and time–frequency domain. Subsequently, irrelevant and redundant features are filtered out, retaining eight key sensitive feature indexes. Finally, using the principal component analysis (PCA) method, these sensitive feature indexes are reduced from high-dimensional space to one dimension and then normalized, thereby constructing a HIVS that can significantly indicate the performance status of the bearings.

A new RUL prediction model, named TCN–Transformer, has been developed to efficiently learn and integrate both local and global features from bearing vibration signals, thereby addressing the challenges associated with long time series predictions in RUL estimation. The model utilizes TCN to integrate signals from different frequency domains, while leveraging the Transformer’s capabilities to process time domain signals, effectively handling the complexities of bearing life evolution.

The subsequent sections of this paper are structured as follows: In Section 2, the methods of feature extraction and HI construction for rolling bearings using vibration signals are proposed, including feature extraction, sensitive feature index selection, dimensionality reduction, and normalization. In Section 3, the RUL prediction model, TCN–Transformer, is proposed, and the RUL process for rolling bearings is introduced. In Section 4, both the HI construction and RUL prediction methods are tested and verified on the IEEE PHM 2012 Data Challenge dataset for RUL prediction of rolling bearings. Finally, conclusions are summarized in Section 5.

## 2. Feature Extraction and Health Index Construction Method

Rolling bearings generate complex vibration signals during operation, which contain a large amount of information about their health status. Through effective feature extraction, key indexes describing the bearing degradation state, such as vibration amplitude, frequency distribution, etc., can be identified from these signals. These indexes can intuitively reflect the working status of the bearing and are important indicators for evaluating bearing performance.

This work first extracts 32 feature indexes in the time domain, frequency domain, and time–frequency domain from the original vibration signals to construct an initial feature set. Then, based on the four feature evaluation indices of monotonicity, correlation, predictability, and robustness, irrelevant and redundant features are filtered out from the original feature set, and eight key sensitive feature indexes that can accurately reflect the performance of rolling bearings are retained. Finally, these sensitive feature indexes are reduced from high-dimensional space to one dimension and then normalized, thereby constructing a HI that can significantly indicate the performance status of the bearings.

### 2.1. Feature Extraction Method

In our previous work [8], it was demonstrated that vibration signals of rolling bearings can be decomposed into multiple characteristic waveforms in the frequency domain through empirical functional decomposition.

As bearing performance degrades over time, both frequency domain and time domain characteristics exhibit temporal variations. Considering that the four primary failure modes of bearings, fatigue pitting, plastic deformation, wear, and cage damage, are mutually independent, selecting several dominant features to characterize this model is of significant importance for reducing model parameters.

1. Time domain feature extraction

Usually, feature extraction methods include three categories: time domain analysis, frequency domain analysis, and time–frequency domain analysis. Time domain features include basic statistics (such as mean, variance, peak, etc.) and advanced statistical indicators (such as skewness, kurtosis, etc.). These features can describe the distribution characteristics and change patterns of vibration signals from different perspectives. However, the reliability of many parameters will decrease as the fault progresses, after reaching a certain level. By extracting and analyzing these time domain features, a bearing HI can be constructed, thereby realizing early identification and prediction of bearing performance degradation. In this work, 10 dimensional and 9 dimensionless time domain features are extracted from the original vibration signals of rolling bearings, as listed in Table 1.

2. Frequency domain feature extraction

Frequency domain feature extraction of rolling bearings is one of the key techniques for understanding and analyzing the health state. By converting the vibration signal from the time domain to the frequency domain, the main frequency components and their amplitudes in the signal can be identified, which is extremely important for detecting specific fault types of bearings. Frequency domain analysis is usually implemented with the help of Fourier transform, which can decompose time series data into a series of frequency components, thereby revealing the characteristic frequencies and harmonics of the working bearings. Frequency domain analysis has obvious advantages over time domain analysis in identifying complex fault modes. It can more accurately distinguish and locate early signs of bearing failure, especially small changes in the background of noise. In this work, five frequency domain features are extracted from the original vibration signals of rolling bearings, as listed in Table 2.

3. Time–frequency domain feature extraction

When the performance of rolling bearings degrades, the energy of each node after wavelet packet decomposition will also change accordingly. Therefore, the wavelet packet energy of the vibration signal after wavelet packet decomposition can be used to select certain specific sub-band energies as characteristic indexes to characterize the degradation of rolling bearings. According to the literature [38], the Haar wavelet is used as the basis function to perform a three-layer wavelet packet decomposition on the vibration signals of the rolling bearing in this work. After decomposition, eight sub-bands are obtained, and the energy ratios of the eight sub-bands are used as the time–frequency feature indexes (S25–S32). The energy of the wavelet packet sub-band is defined as follows:

(1) $$E_{j}^{i}\left(t\right)=\sum_{n=1}^{N}\left[x_{jn}^{i}{\left(t\right)}^{2}\right]$$

where $E_{j}^{i}\left(t\right)$ is the wavelet packet energy. *N* is the length of the node signal $x_{j}^{i}\left(t\right)$ after wavelet packet decomposition.

The wavelet packet energy ratio reflects the performance degradation of the rolling bearing by calculating the energy ratio of the wavelet packet reconstructed signal at different time scales. The energy ratio $p_{j}^{i}$ of each sub-band after wavelet packet decomposition is defined as follows:

(2) $$p_{j}^{i}=\frac{E_{j}^{i}(t)}{\sum_{j}E_{j}^{i}(t)}$$

### 2.2. Constructing the Sensitive Feature Set for Rolling Bearings

The goal of feature selection is to find a set of feature subsets that are effective for performance assessment, which can ensure that the prediction performance is maintained at a good level while the feature dimension is reduced. The purpose of constructing a sensitive feature set is to select the features that are most sensitive to changes in the bearing state and best reflect its health status from a large number of original or derived features. It can not only significantly reduce the dimensionality of data and reduce the computational task of model training but also improve the robustness and interpretability of prediction models. By eliminating redundant and irrelevant features, feature selection helps focus on those most representative indexes, thereby providing a more accurate assessment of bearing performance. This work defines four evaluation indices for effective feature selection, namely monotonicity index, correlation index, predictive index, and robustness index, and establishes the process of selecting optimal features as a combinatorial optimization problem to construct sensitive feature set for rolling bearings.

Monotonicity refers to the unidirectional change tendency of features as performance degrades. This work uses Spearman’s rank correlation coefficient as the monotonicity index [39], and the formula is as follows:

(3) $$Mo(Y)=\left|1-\frac{6\sum_{i=1}^{N}d_{i}^{2}}{N(N^{2}-1)}\right|$$

where *d* is the difference between the two variables, and *N* is the total number of monitoring times during the entire performance degradation process.

Temporal correlation emphasizes the dependence of features on time series. This work uses the Pearson correlation coefficient to describe the correlation between features and time series. The formula is as follows:

(4) $$Mo(Y)=\left|1-\frac{\left|6\sum_{i=1}^{N}\left(y_{i}-\bar{y}\right)\left(t_{i}-\bar{t}\right)\right|}{\sqrt{\sum_{i=1}^{N}{\left(y_{i}-\bar{y}\right)}^{2}\sum_{i=1}^{N}{\left(t_{i}-\bar{t}\right)}^{2}}}\right|$$

where $Y=[y_{1},y_{2},\ldots ,y_{N}]$ is the performance feature sequence, $t_{i}$ represents the i-th monitoring moment, $\bar{t}$ and $\bar{y}$ represent their means. *N* is the total number of monitoring times in the entire performance process.

Predictability means that the features can provide enough information to predict the future state. Predictive index is defined as follows:

(5) $$Pre(Y)=\exp\left(-\frac{\sigma (y_{f})}{\left|{\bar{y}}_{f}-{\bar{y}}_{s}\right|}\right)$$

where *y* is the performance characteristic, ${\bar{y}}_{s}$ is the mean at the initial moment, ${\bar{y}}_{f}$ is the mean at the failure moment, and $\sigma (y_{f})$ is the standard deviation at the failure moment.

Robustness refers to the ability of a feature set to maintain its predictive performance in the face of various disturbances and noise. Robustness index is adopted as follows:

(6) $$Rob(Y)=\frac{1}{N}\sum_{n=1}^{N}\exp\left(-\left|\frac{y_{i}-{\tilde{y}}_{i}}{y_{i}}\right|\right)$$

where $Y=[y_{1},y_{2},\ldots ,y_{N}]$ is the performance feature sequence, *N* is the total number of monitoring times in the entire performance process, and $Y=\left[{\tilde{y}}_{1},{\tilde{y}}_{2},\ldots ,{\tilde{y}}_{N}\right]$, is the trend sequence of the corresponding performance feature.

Relying only on a single evaluation indicator to evaluate the features is often incomplete for performance degradation assessment. To fully utilize multiple evaluation indices, and assuming that the discrete features can be approximated linearly, a weighted linear combination model integrating multiple evaluation indices is constructed to determine performance degradation features, in view of the important role of monotonicity, correlation, predictability, and robustness in the performance prediction of rolling bearings. A comprehensive index is introduced and defined as follows:

(7) $$\begin{array}{l} J=w_{1}Mon(Y)+w_{2}Corr(Y,T)+w_{3}Pre(Y)+w_{4}Rob(F),\;w_{i}>0\\ \sum_{i}w_{i}=1,i=1,2,3,4 \end{array}$$

For the characterization and prediction of the performance of rolling bearings, the key is to select features that reflect the overall degradation trend because the degradation of rolling bearings is monotonic and irreversible. Therefore, in the comprehensive index, the importance of monotonicity should be fully considered, and its weight is relatively high. Performance degradation is a continuous process, and its changes show certain regularity in time series. Considering temporal correlation can help understand the dynamic degradation process and extract features that can reflect the degradation trend, thereby improving the time sensitivity and accuracy of the prediction model. However, in experiments, it was found that the extracted features usually have higher robustness, resulting in a decrease in robustness discrimination, so the weight is lower. By referring to the parameter settings and experimental results in the literature, the weights of $w_{1},w_{2},w_{3},w_{4}$ are set to 0.4, 0.4, 0.1, and 0.1, respectively [40].

### 2.3. Dimensionality Reduction Method for Sensitive Feature Index

PCA, as a widely adopted statistical technique, can be used for data dimensionality reduction and feature extraction [41]. It converts multiple variables that may be correlated into a series of linearly independent variables through orthogonal transformation. These new variables are called principal components. In the application of dimensionality reduction in sensitive feature indexes, PCA shows significant advantages. By using PCA to reduce dimensionality, the complexity of the model can be effectively reduced, the computing efficiency can be improved, while overfitting can be avoided, and the generalization ability of the model can be enhanced.

**Figure 1:**
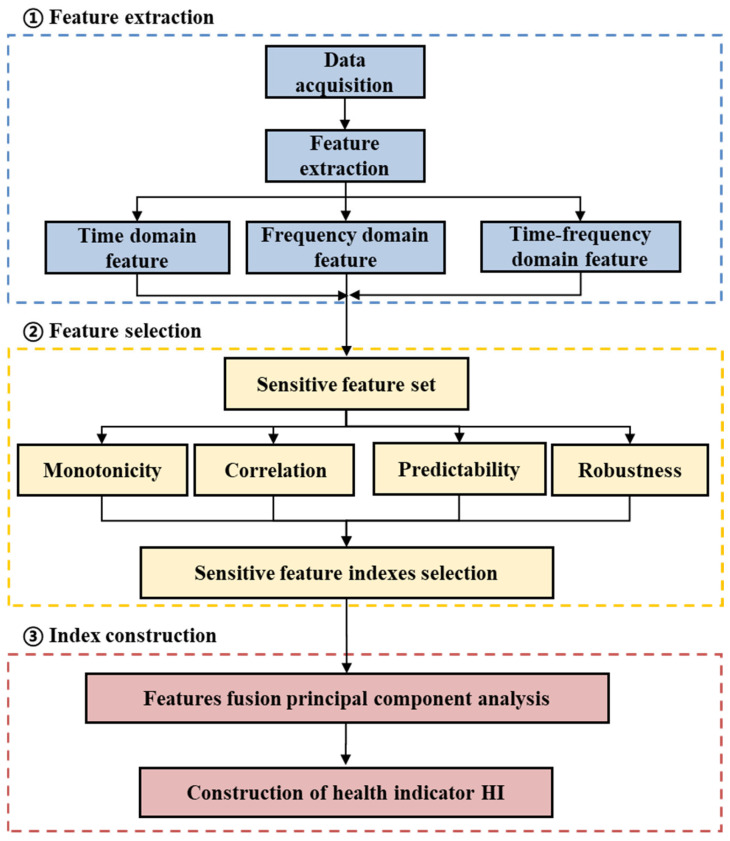
Flowchart of dimensionality reduction in sensitive feature indexes based on principal component analysis method.

The dimensionality reduction process of sensitive feature indexes based on PCA is shown in Figure 1, and the details are as follows:

1. Feature extraction of vibration signals. There are 32 feature indexes used here, namely 10 dimensionless time domain indexes (S1–S10), 9 dimensionless time domain indexes (S11–S19), 5 frequency domain characteristics (S20–S24), and 8 time–frequency domain indexes (S25–S32), regarding the energy ratio of sub-bands. Using the original vibration signal, a total of 32 feature indexes above are extracted to form the original feature set.
2. Sensitive feature index selection based on evaluation indices. Based on the comprehensive index that takes into account the monotonicity, correlation, predictability, and robustness of the features, eight sensitive feature indexes of rolling bearings are selected.
3. Dimensionality reduction in sensitive feature indexes. The selected sensitive degradation features are input into the PCA algorithm as input data, and the first principal component is extracted as the rolling bearing performance degradation feature index after dimensionality reduction.

### 2.4. Constructing the Health Index for Rolling Bearing

After selecting, the eight feature indexes are spliced into a 2D array by column, in which each column represents a feature vector. This 2D array is input into the PCA algorithm as input data, and the first principal component is extracted as the performance degradation feature index of rolling bearing after dimensionality reduction. Then, the obtained performance feature index is processed to remove outliers and then normalized by min–max normalization approach to obtain its health index. Finally, the health index is smoothed by the moving average method to obtain the final performance degradation trend of the rolling bearing. The normalization formula is as follows:

(8) $$x_{\mathrm{nor}}=\frac{x-x_{\min}}{x_{\max}-x_{\min}}$$

where $x_{\mathrm{nor}}$ is the normalized value, and $x_{\max}$ and $x_{\min}$ are the maximum and minimum values of this group of sequences, respectively. The moving average formula is as follows:

(9) $$y_{t}=\frac{\sum_{i=1}^{n}\left(x_{t-i}+x_{t+i}\right)+x_{t}}{2n+1}$$

where $y_{t}$ is the smoothed value obtained at time *t*. $x_{t}$ is the value of original sequence at time *t*, and *n* is the window size of sliding average.

## 3. TCN–Transformer Networks and RUL Prediction

As a structural innovation model based on CNN, TCN achieves overall parallel processing capabilities for long-term sequences by using a unified filter in each layer. Compared with traditional recursive network models, such as LSTM and GRU, TCN is more concise and clearer in structural design, while also improving accuracy [35]. TCN can effectively adjust the size of the receptive field by stacking more causal convolution layers, increasing the expansion factor, and increasing the number of filters, thereby flexibly controlling the memory usage of the model. Faced with the common problem of gradient explosion or disappearance in RNN, TCN effectively avoids this problem with its unique backpropagation path and ability to handle different sequence times. In addition, TCN can significantly shorten the training cycle due to its low memory requirements when processing long-term sequences.

Given the Transformer’s limitations in effectively utilizing information across multi-parameter temporal domains, this study adopts two key strategies: (1) manual selection of critical feature parameters, and (2) processing of cross-feature temporal variations through a dedicated TCN module. In this architecture, the Transformer’s role is specifically focused on applying attention mechanisms to perform RUL (remaining useful life) prediction using these processed features.

1. Causal convolution

TCN needs to meet two major requirements: (1) ensuring that the network output length is consistent with the input length, using a one-dimensional fully convolutional network (FCN), and keeping the length of each layer unchanged by zero padding; (2) ensuring that future inputs will not affect past inputs, which is achieved by using causal convolution to eliminate the interference of future elements, as the output at any time t is only related to the current and previous input elements.

2. Dilated convolution

When processing historical data, causal convolution requires more hidden layers as the depth of historical data increases, which requires a deeper network structure or more filters. To solve this problem, dilated convolution technology was introduced [24]. By adding holes to standard convolution, dilated convolution can effectively expand the size of the receptive field, so that the output data can cover a wider range of information without losing data in the pooling layer.

3. Residual module

**Figure 2:**
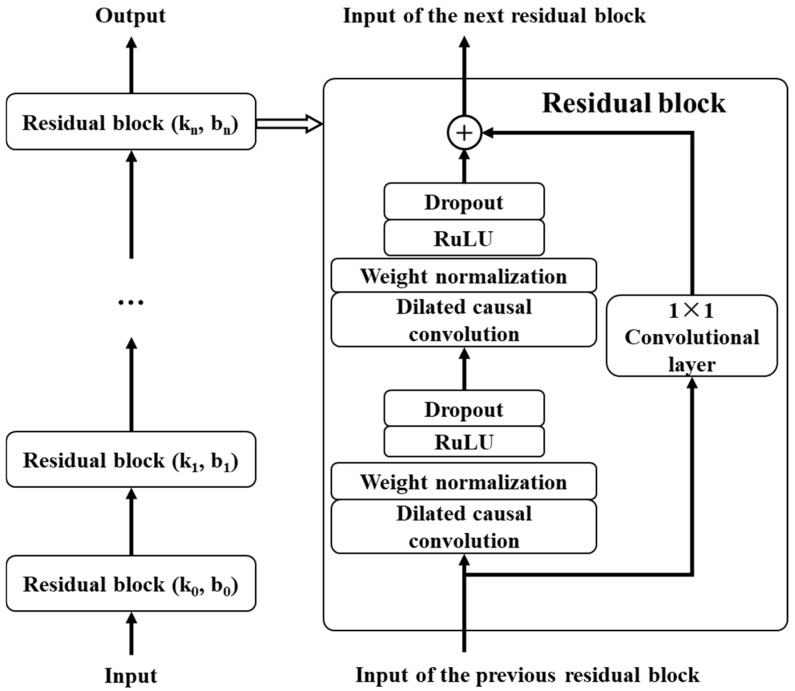
Framework of TCN model.

In order to solve the performance degradation problem caused by the increase in network depth, the concept of residual module is proposed. In the residual module, the rectified linear unit (ReLU) is used as the activation function, and the weight normalization method is used to normalize the weight of the convolution filter for normalization. At the same time, in order to further enhance the generalization ability of the model, a spatial dropout step is added for regularization after each dilated convolution operation, that is, the output of the entire channel is randomly set to zero in each step of the training process. In addition, to deal with the shape mismatch problem between input and output, an additional 1 × 1 convolution layer is introduced in TCN to ensure that tensors of the same shape can be transferred between different layers, as shown in Figure 2.

### 3.1. Construction of TCN–Transformer Networks

**Figure 3:**
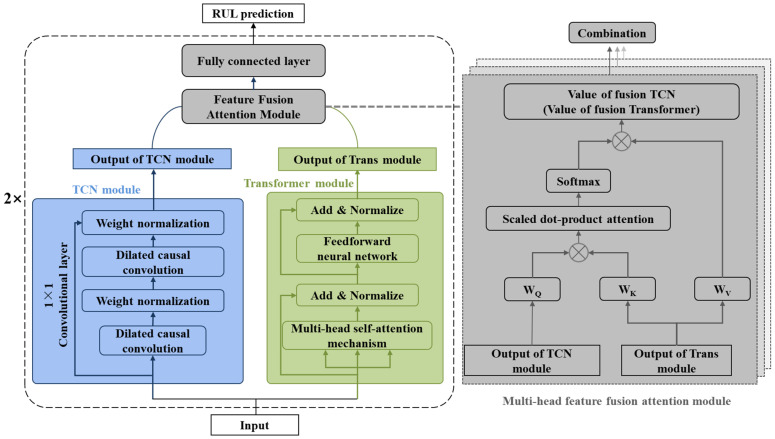
Framework of proposed TCN–Transformer networks.

The TCN–Transformer architecture employs a hierarchical parallelization approach to integrate TCN and Transformer components. In the design, the TCN layer extracts local temporal features from the input sequence, while the Transformer module captures global data patterns. These learned representations are subsequently combined using a multi-head feature fusion attention module. The parallel structure concludes by concatenating both branches’ outputs, followed by a fully connected layer that projects them to the target dimension while maintaining the original time series structure. Compared with conventional TCN and Transformer, TCN–Transformer introduces two key innovations: (1) a hierarchical parallel architecture that simultaneously utilizes the Transformer block’s local window self-attention and the TCN block’s deep convolutional operations; (2) the incorporation of a specialized multi-head features fusion attention module for effective branch feature integration. Figure 3 illustrates the framework of the TCN–Transformer network.

(1) Hierarchical parallel design

As shown in Figure 3, the TCN–Transformer network comprises parallel computation flows for both the Temporal Convolutional Network (TCN) and the Transformer (Trans) modules. Inside the TCN flow, there are two dilated causal convolution layers with weight normalization, which constitute a hidden layer of the TCN model. The input of the TCN layer is the data after the rolling bearing vibration signal is preprocessed or the features of the local and global features fused from the previous layer, and the output of the TCN layer is the local features of the bearing signal. The Transformer module has a multi-head self-attention mechanism and a feedforward neural network, both equipped with layer normalization. Similarly, the input to this module comprises the preprocessed rolling bearing vibration signal data, which includes the fused local and global features from the prior layer. The output of the Transformer module captures the global features of the bearing’s full life vibration signal [42].

(2) Multi-head feature fusion attention module

The multi-head feature fusion attention module contains two attention mechanisms, which aims to establish an interaction between two parallel branches to fuse local and global features. As shown on the right side of Figure 3, the output of the TCN layer $\tilde{Y_{i}}\in R^{t\times c}$ and the output of the Transformer module ${\tilde{Z}}_{i}\in R^{t\times c}$ interact in the multi-head feature fusion attention module to bidirectionally fuse local features $\tilde{Y_{i}}$ and global features $\tilde{Y_{i}}$. Specifically, the output value of the TCN layer is updated by residual connection with the multi-head feature fusion attention module to obtain the new TCN layer output value $Y_{i+1}$, as described below:

(10) $$Y_{i+1}={\tilde{Y}}_{i}+A_{{\tilde{Z}}_{i}\to {\tilde{Y}}_{i}}\cdot Z_{i}W_{ev}$$

where $W_{ev}$ is the learnable parameter of embedding layer, and $A_{{\tilde{Z}}_{i}\to {\tilde{Y}}_{i}}$ is the fusion matrix from Transformer to TCN, which can be calculated by matrix multiplication and Softmax function:

(11) $$A_{{\tilde{Z}}_{i}\to {\tilde{Y}}_{i}}=softmax(\frac{{\tilde{Y}}_{i}W_{eq}\cdot {\left({\tilde{Z}}_{i}W_{ek}\right)}^{T}}{\sqrt{c}})$$

where $W_{eq}$ and $W_{eq}$ are the learnable parameters of the two linear layers. Similarly, the output value of the updated Transformer module is defined as follows:

(12) $$Z_{i+1}={\tilde{Z}}_{i}+A_{{\tilde{Y}}_{i}\to {\tilde{Z}}_{i}}\cdot Y_{i}W_{dv}$$

(13) $$A_{{\tilde{Y}}_{i}\to {\tilde{Z}}_{i}}=softmax(\frac{{\tilde{Z}}_{i}W_{dq}\cdot {\left({\tilde{Y}}_{i}W_{dk}\right)}^{T}}{\sqrt{c}})$$

where $W_{dv}$, $W_{dq}$, and $W_{dk}$ are the learnable parameters of the three linear layers. $A_{{\tilde{Y}}_{i}\to {\tilde{Z}}_{i}}$ is the fusion matrix from TCN to Transformer.

### 3.2. RUL Prediction Based on TCN–Transformer

**Figure 4:**
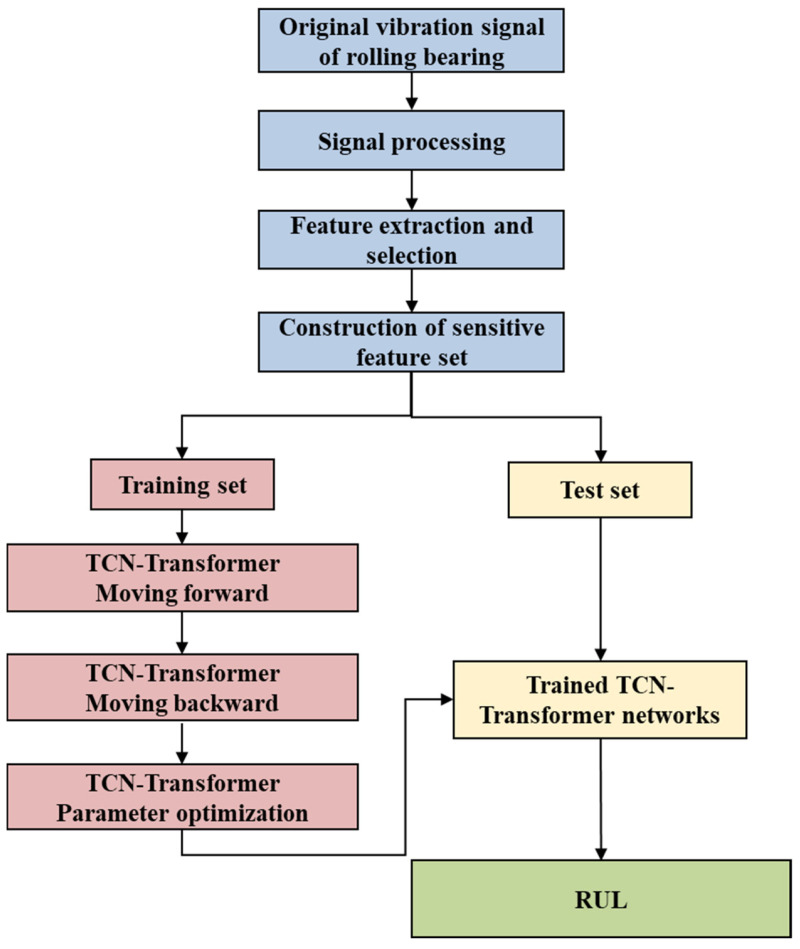
The remaining useful life prediction process of rolling bearings based on the TCN–Transformer networks.

This section describes the RUL prediction process of rolling bearing based on the TCN–Transformer networks in detail. The flowchart is shown in Figure 4. The specific steps are as follows:

(1) Data input. The original vibration signal data of the rolling bearing is processed. According to the method proposed, the original vibration signal is extracted in the time domain, frequency domain, and time–frequency domain. Subsequently, sensitive features are selected to construct a feature set. The selected sensitive degradation feature data is input into the model to train the model for remaining life prediction.
(2) Dataset division. Referring to the most commonly used dataset division method, the dataset is divided into training set, validation set, and test set in a ratio of 7:1:2.
(3) Model training. The training set data is input into the constructed TCN–Transformer networks. TCN–Transformer trains the model and completes the steps of forward propagation, backpropagation, and parameter optimization. The TCN–Transformer network with the optimal parameters is obtained.
(4) Model prediction. Input the test set data into the optimal model trained in the third step and finally output the RUL prediction result of the rolling bearing.

## 4. Results and Discussion

### 4.1. Verification of Feature Extraction and Health Index Construction

The IEEE PHM 2012 Data Challenge published a rolling bearing full lifecycle dataset, which collected on the PRONOSTIA experimental system that was designed to test bearing fault detection, diagnosis, and RUL prediction methods [43]. The main goal of PRONOSTIA is to provide experimental data to describe the degradation process of rolling bearings throughout their service life. The IEEE PHM 2012 dataset provides accelerated degradation test data for a total of 17 bearings, namely, 7 bearings for condition 1 (4000 N, 1800 rpm), 7 bearings each for condition 2 (4200 N, 1650 rpm), and 3 bearings for condition 3 (5000 N, 1500 rpm). In this work, all the 17 bearings were used as a benchmark to test our prediction method, but only the results for seven bearings under condition 1, marked as Bearing 1-1, 1-2, 1-3, 1-4, 1-5, 1-6, and 1-7, respectively, were selected for further investigation. When using vibration signals to track the degradation state of rolling bearings, the horizontal vibration signal usually carries more degradation information than the vertical vibration signal [44]. Therefore, this work only uses horizontal experimental data.

**Figure 5:**
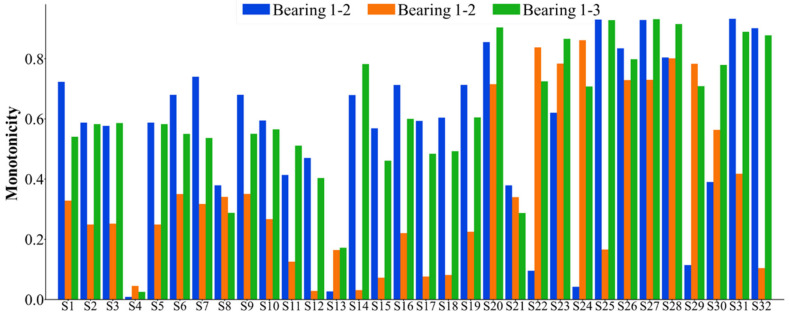
A statistical chart of the monotonicity index of the 32 feature indexes.

**Figure 6:**
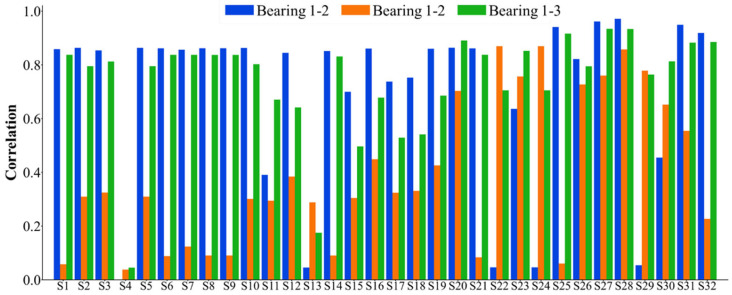
A statistical chart of the correlation index of the 32 feature indexes.

**Figure 7:**
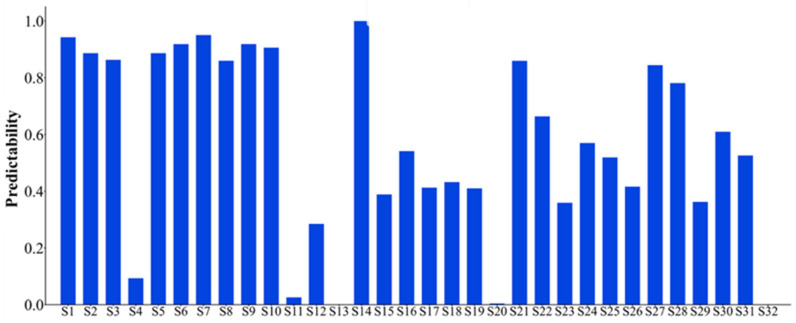
Statistical chart of predictive index of 32 feature indexes.

**Figure 8:**
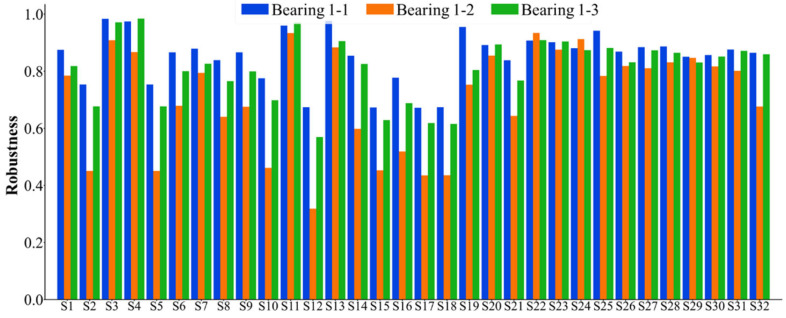
A statistical chart of the robustness index of the 32 feature indexes.

**Figure 9:**
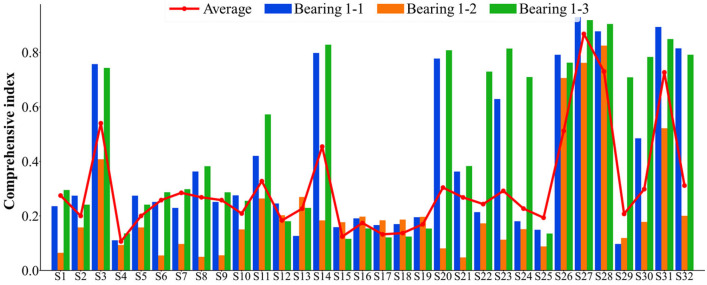
Statistical chart of comprehensive indexes of 32 feature indexes.

The 32 feature indexes introduced above are extracted to form the original dataset for the seven bearings. Subsequently, these 32 indices are selected accordingly. Considering that the failure vibration signals of the bearings can be represented by eight wave packets, the assessment of bearing failure utilizes the 32 indices, which are comprehensive in nature. For the dataset of each bearing, the monotonicity index, correlation index, predictive index, robustness index, and comprehensive index are calculated. The statistical results of the feature indexes are shown in Figure 5, Figure 6, Figure 7, Figure 8 and Figure 9. Only the data of bearings 1-1, 1-2, and 1-3 are shown in the charts. In particular, Figure 9 shows the comprehensive index statistics of the three bearings, and also gives the average values of the comprehensive index of the seven bearings under working condition 1.

**Figure 10:**
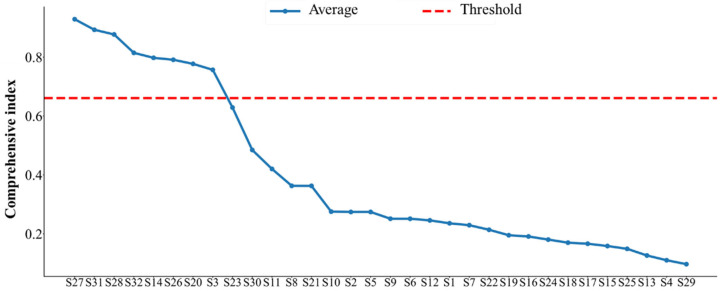
Ranking of the values of the comprehensive indexes of the 32 feature indexes.

**Figure 11:**
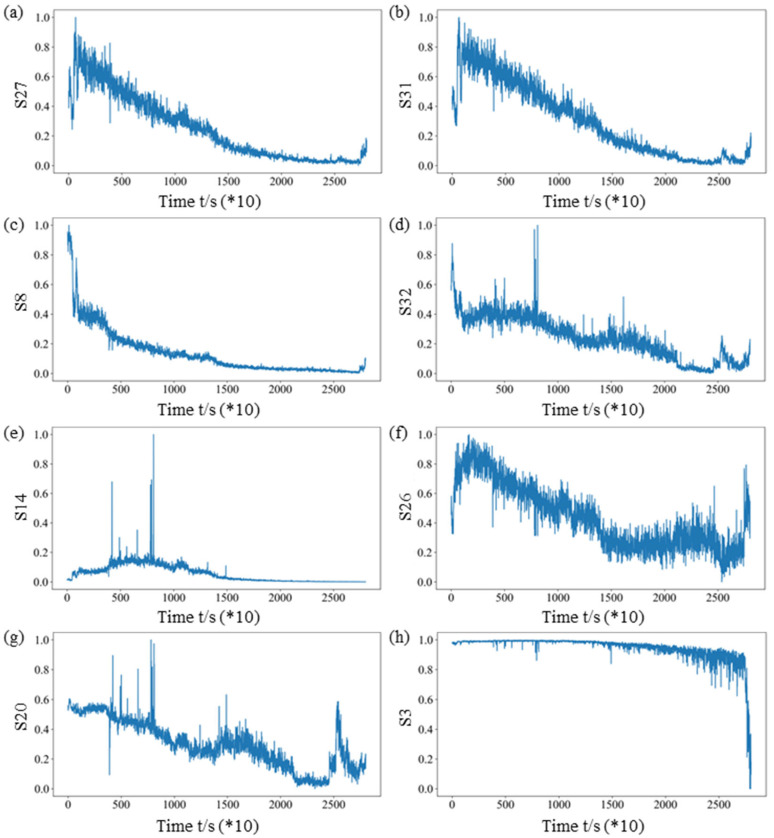
The selected sensitive feature indexes for Bearing 1-1: (**a**) the energy ratio of the third frequency sub-band (S27), (**b**) the energy ratio of the seventh frequency sub-band (S31), (**c**) the energy ratio of the fourth frequency sub-band (S28), (**d**) the energy ratio of the eight frequency sub-band (S32), (**e**) the kurtosis index (S14), (**f**) the energy ratio of the second frequency sub-band (S26), (**g**) the center of gravity frequency (S20), and (**h**) the minimum value (S3).

The feature indexes are sorted according to the average value of the comprehensive index, as shown in Figure 10. Only the eight feature indexes with the highest average value of the comprehensive index are selected, which are the energy ratio of the third frequency sub-band (S27), the energy ratio of the seventh frequency sub-band (S31), the energy ratio of the fourth frequency sub-band (S28), the energy ratio of the eight frequency sub-band (S32), the kurtosis index (S14), the energy ratio of the second frequency sub-band (S26), the center of gravity frequency (S20), and the minimum value (S3), respectively. To make a further demonstration, the degradation trend of the eight selected sensitive feature indexes over time is shown in Figure 11.

**Figure 12:**
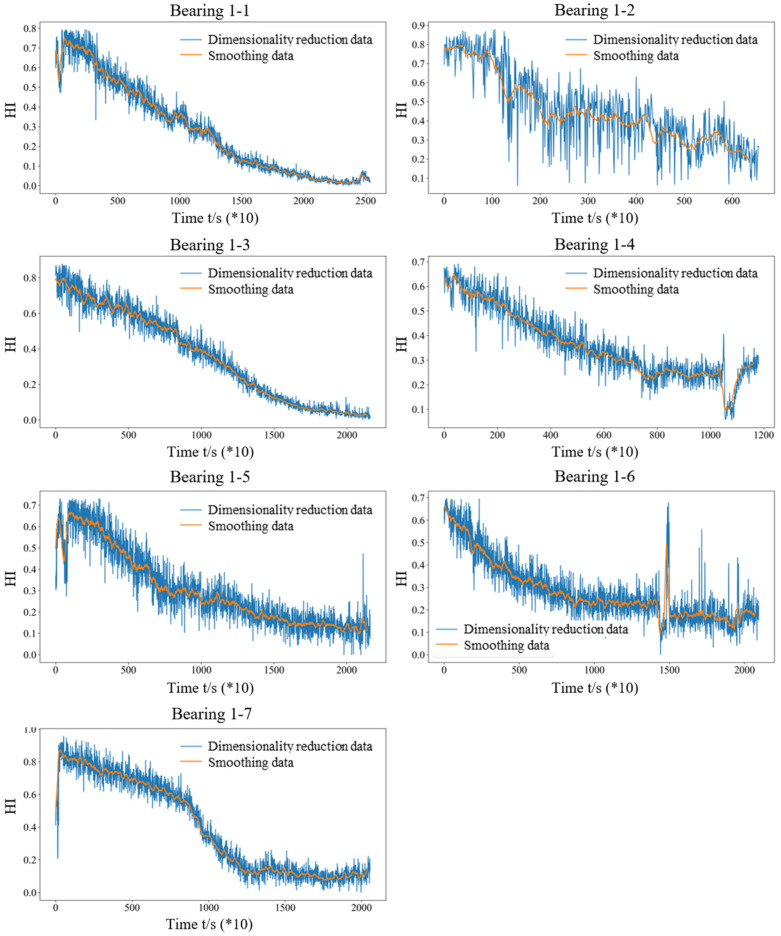
The health index trend over time of the seven bearings obtained using the proposed method.

Figure 12 shows the health index trend of the seven bearings obtained using the feature extraction, selection, and PCA dimensionality reduction methods. It can be seen that the health index of rolling bearings obtained based on the proposed method in this work has good monotonicity and time series correlation. Subsequently, the monotonicity, correlation, predictability, robustness, and comprehensive indexes of the selected feature indexes for the seven datasets are calculated, as given in Table 3. It can be seen that the obtained monotonicity, correlation, predictability, robustness, and comprehensive indexes are relatively good, all above 0.8. Meanwhile, the PCA result indicates that the seven main features can account for 91.387% of the variance in the data. For industrial problems, such explanatory power is sufficient. In addition, all the comprehensive indexes obtained in this work are all higher than the highest original comprehensive index for each feature set. The average comprehensive index of the seven bearings is 0.8995, improved by 8.69% on average.

### 4.2. Experiment and Verification of the TCN–Transformer Networks

This section uses the IEEE PHM 2012 rolling bearing full life dataset as the verification dataset for the proposed method. The detailed information of the dataset used in this section is shown in Table 4. It shows information such as working conditions and actual life. In order to better verify the effectiveness of the proposed method, this paper refers to the task setting of previous literature [45] and carefully sets up six groups of RUL estimation tasks to evaluate the prediction performance of the proposed method. The specific arrangement is shown in Table 5.

The training of the neural network based on 32 feature indexes was conducted on an Ubuntu 18.04 server equipped with four NVIDIA 2080 Ti 11 GB graphics cards, using PyTorch 1.0 as the deep learning framework.

First, data preprocessing should be carried out in the analysis. While deep learning models inherently can extract features directly from raw vibration signals (an approach widely adopted in PHM applications), the high computational demands and compromised prediction accuracy associated with processing full lifecycle raw data make this method impractical. To address these challenges in long-sequence RUL prediction, we employ the optimized eight-feature degradation set as the model input, achieving significant reduction in data dimensionality while preserving degradation signatures, and elimination of noise interference in raw signals. This preprocessing strategy balances computational efficiency with predictive performance.

Next, RUL labeling and normalization are implemented to standardize the prognostic framework. Since operational conditions vary significantly, the absolute RUL values (measured in cycles) naturally differ in magnitude. Direct use of these unprocessed values as training labels would adversely affect both the model’s convergence rate during training and its generalization performance during deployment. To address this, we adopt normalized RUL values—a well-established practice in prognostic modeling [46]. The normalization process computes the relative degradation state by taking the ratio of current RUL to the entire RUL value, expressed as follows:

(14) $$RUL_{t}=T_{total}-t$$

(15) $$RUL_{t}^{norm}=\frac{RUL_{t}}{T_{total}}$$

where $T_{total}$ is the total life time, and the normalized remaining life $RUL_{t}^{norm}$ is between 0 and 1.

Effective sample embedding obviously influences the predictive capability of data-driven prognostic models. Utilizing isolated single-time step data as model input fails to capture the essential temporal dependencies between current and historical degradation states. To address this limitation, we implement a temporal window embedding strategy [46] that explicitly models degradation continuity through causal relationships. This method employs a fixed time window *W*_L_ to sequentially concatenate multiple time steps of monitoring data and treats each window as an independent input. The embedded sample consists of the current time step *MS* and the previous *L* − 1 time step $MS_{s}$, noted as follows:

(16) $$MS_{Input}^{t}={\left(MS_{t-L+1},\ldots ,MS_{t-2},MS_{t-1},MS_{t}\right)}_{W_{L}}$$

where *L* is the time window length. In order to obtain as many training samples as possible, the moving step of the time window is generally set to 1.

In order to make a comparison with the existing advanced prediction models, three criteria of root mean square error (RMSE), mean absolute error (MAE), and scoring function (SCORE) are used to evaluate the prediction performance. Among them, the first two can evaluate well the fitting ability and prediction accuracy of each prediction model, and the calculation formulas are as follows:

(17) $$RMSE=\sqrt{\frac{1}{n}\sum_{i=1}^{n}({\hat{y}}_{i}-y_{i}{)}^{2}}$$

(18) $$MAE=\frac{1}{n}\sum_{i=1}^{n}\left|{\hat{y}}_{i}-y_{i}\right|$$

where ${\hat{y}}_{i}$ is the predicted value at time *i*, $y_{i}$ is the true value at time *i*, and *n* represents the total number of samples. In addition, the last evaluation criterion is used to evaluate the rationality of the prediction results, that is, the proactive prediction and the lagging prediction are not considered with a unified standard. In the actual service environment, the risk brought by the advanced prediction is much smaller than that of the lagging prediction. Therefore, a good prediction RUL tends to be a conservative prediction, so this scoring algorithm imposes a penalty on the lagging prediction. Therefore, SCORE can be defined as follows [43]:

(19) $$SCORE=\frac{1}{N-1}\sum_{i=1}^{N-1}A_{i};A_{i}=\left\{\begin{array}{l} e^{-\ln(0.5)\cdot (Er_{i}/5)},Er_{i}\leq 0\\ e^{+\ln(0.5)\cdot (Er_{i}/20)},Er_{i}>0 \end{array}\right.$$

(20) $$Er_{i}=\frac{y_{i}-{\hat{y}}_{i}}{y_{i}}\times 100\%$$

where *N* is the length of the prediction data, and $Er_{i}$ is the percentage error.

In order to further clarify the advantages of TCN–Transformer in RUL prediction, this work makes two comparisons. One is to compare the RUL prediction performance of TCN–Transformer networks, TCN model, and Transformer model, and the other is to compare the proposed model with several baseline models and advanced prediction models. To explore the degree of improvement of the TCN–Transformer networks on each task, the improvement index (IMP) is calculated as follows:

(21) $$IMP=\left(1-\frac{TT}{CMT}\right)\times 100\%$$

wherein *TT* represents the evaluation index value of the TCN–Transformer networks, and CMT represents the evaluation index value of the current optimal model. During the model training process, the settings of various hyperparameters are shown in Table 6. At the same time, 10 cross-validation experiments are performed, where 70% of the data is used for training, 10% for validation, and 20% for testing. Each task obtained the average results to avoid the randomness of the prediction results.

**Figure 13:**
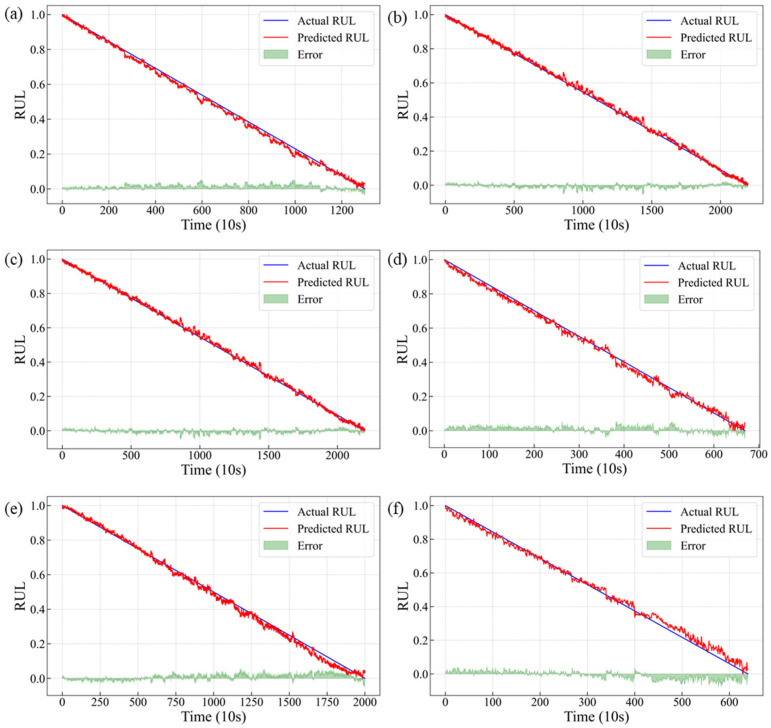
RUL prediction results for rolling bearings: (**a**) Task A; (**b**) Task B; (**c**) Task C; (**d**) Task D; (**e**) Task E; (**f**) Task F.

Figure 13 displays the comparative results between predicted and actual RUL values for the six tasks, including detailed error analysis. The TCN–Transformer demonstrates remarkable tracking capability, with its prediction curve maintaining close proximity to the truth RUL, thereby successfully capturing the bearing’s degradation characteristics and providing initial validation of the model’s efficacy. Particularly noteworthy is the model’s superior prediction performance during approximately 80% of the lifespan, while most deviations tend to emerge in the final degradation stage. This may originate from the traditional linear degradation assumption in RUL prediction, which is valid during stable operation. However, actual failure process often exhibits complex nonlinear behavior, especially during accelerated deterioration stages where degradation rates follow exponential growth patterns. Simultaneously, during the advanced stages of degradation, significant variations in degradation rates are observed among different bearings, indicating a discrepancy between the assigned training labels and actual degradation states. The prediction errors of our model predominantly occur in the later degradation stages. Furthermore, the cumulative effect of these errors amplifies the observed divergence.

**Figure 14:**
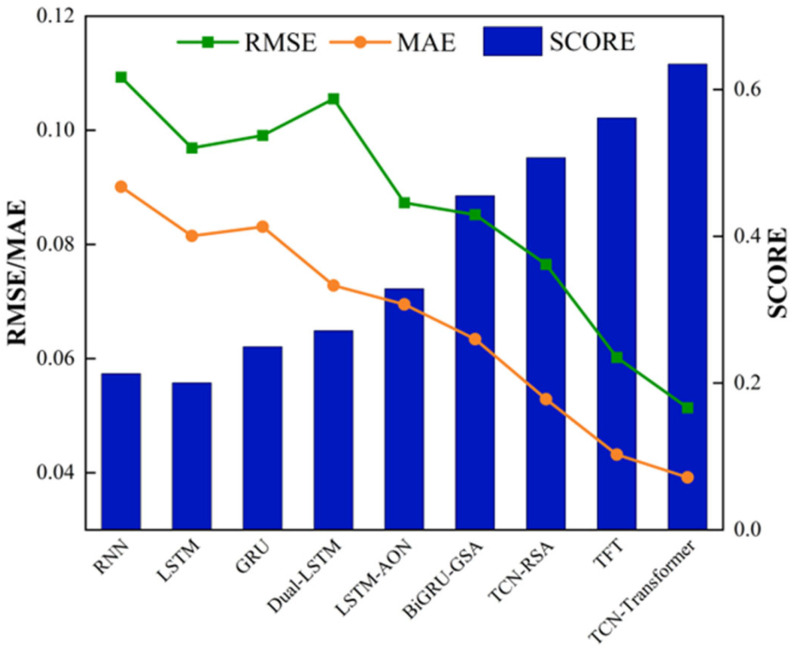
Comparison of evaluation indices of TCN–Transformer and other models.

Table 7 shows the comparison between TCN–Transformer networks and other advanced models. The baseline models compared here include RNN, LSTM, and GRU, and the five most advanced prediction models include Dual-LSTM [47], LSTM-AON [48], BiGRU-GSA [49], TCN-RSA [49], and TFT [45]. Compared with the prediction results of baseline and state-of-the-art models (even including the current best-performing TFT model), the TCN–Transformer networks proposed in this work achieved the best evaluation index results. The IMP of RMSE, MAE, and SCORE are 14.62%, 9.26%, and 13.04%, respectively, which shows that the proposed model is more competitive in RUL prediction. Specifically in terms of the rationality of RUL prediction, the SCORE of the TCN–Transformer networks is significantly higher than other models, which is the key to evaluating the reliability of RUL prediction. Figure 14 is a visual comparison between TCN–Transformer and other models, which more intuitively shows that the model proposed has the best results in the three evaluation indices. The above comparative analysis further verified the advantages of TCN–Transformer.

### 4.3. Ablation Experiment and Results of TCN–Transformer Networks

The TCN–Transformer networks are compared with the TCN model and Transformer model on six tasks. Table 8 shows the results of TCN–Transformer networks ablation experiment. Table 5 presents the composition of the experimental dataset. It can be seen that, compared with the TCN model and the Transformer model, the prediction effect of the TCN–Transformer networks has improved on all tasks. The average IMP of RMSE, MAE, and SCORE reached 39.67%, 38.07%, and 26.63%, respectively, which shows that the proposed TCN–Transformer networks can obviously improve the accuracy and rationality of RUL prediction. In addition, the IMP degree of RMSE and MAE is significantly higher than that of SCORE, which indicates that the RUL prediction curve of the proposed TCN–Transformer networks is more consistent with the real RUL curve. The comparison proves that the proposed TCN–Transformer network is superior to the original TCN model and Transformer model, and also proves that the hierarchical parallel design of the TCN–Transformer networks and the multi-head feature fusion attention module are reasonable and effective.

The main purpose of this paper is to develop an advanced technical framework to optimize the performance monitoring and RUL prediction of rolling bearings, contributing to the health monitoring and maintenance methods of key mechanical components in intelligent manufacturing. First, by extracting and selecting key features, a HI set that can accurately reflect bearing performance degradation is constructed, effectively solving the problem of characterizing the performance degradation state. Second, by combining the TCN model and Transformer model, TCN–Transformer networks are proposed, which can efficiently learn and integrate local features and global features, providing a new solution for RUL prediction. These methods not only demonstrate the great potential of deep learning in complex system monitoring and prediction but also provide support for maintenance decisions in practical applications, especially in the field of preventive maintenance. In addition, these methods have broad application prospects which are not limited to rolling bearings or rotating machinery. They can also be extended to performance monitoring and life prediction of other key industrial components, thereby bringing a wider impact to the field of intelligent manufacturing.

Based on the innovative points of this work, there will be more future research focused on aspects such as the mathematical interpretability of the model, parameter optimization, and improvement of the performance of individual modules. Meanwhile, the frequency of the sensor is also of great significance for the performance study of the model. This requires a more systematic experimental platform and data analysis.

## 5. Conclusions

In this work, a method for constructing a HIVS is first developed to describe the performance features of rolling bearings. And then, a new RUL prediction model, TCN–Transformer, was developed to efficiently solve the long time series prediction problem in RUL prediction. The conclusions are summarized as follows:

1. The method for constructing a HIVS was developed to describe the performance features of rolling bearings. The eight sensitive feature indexes that can accurately reflect the performance of rolling bearings were selected from the 32 indexes to construct the feature set, and then the obtained sensitive feature index after dimensionality reduction was processed to remove outliers and then normalized to obtain the HI. The average comprehensive index of bearings improved by 8.69% on average.
2. The TCN–Transformer employs a hierarchical parallel architecture combining TCN and Transformer modules, achieving higher computational efficiency and a more compact network scale. Compared with classical standalone TCN or Transformer networks, our approach significantly reduces the required number of channels through feature compression. The outputs from the TCN and Transformer modules interact through a novel multi-head feature fusion attention mechanism, enabling bidirectional integration of local temporal patterns (captured by TCN) and global dependencies (learned by Transformer). This specialized attention module dynamically prioritizes the most discriminative features extracted by both sub-networks, ensuring precise focus on performance-critical characteristics for RUL prediction.
3. Compared with existing methods, the proposed TCN–Transformer demonstrates superior accuracy in predicting the RUL of rolling bearings across diverse operating conditions. Specifically, in ablation studies, TCN–Transformer outperforms both the standalone TCN and Transformer models, achieving consistent improvements across all evaluation tasks. When compared with state-of-the-art methods, TCN–Transformer reduces RMSE and MAE by 14.62% and 9.26%, respectively, while improving the SCORE metric by 13.04%. These results conclusively validate the superiority of our approach in RUL prediction.

## Figures and Tables

**Table 1:** Dimensional and dimensionless time domain feature indexes used in this work.

Dimensional Index	Function	Dimensionless Index	Function
Mean absolute value (S1)	$x_{av}=\frac{1}{N}\sum_{i=1}^{N}\left|x_{i}\right|$	Skewness (S11)	$x_{ske}=\frac{\sum_{i=1}^{N}{\left(x_{i}-\bar{x}\right)}^{3}}{\left(N-1\right)x_{\sigma }^{3}}$
Peak (S2)	$x_{p}=\max|x_{i}|$	Kurtosis (S12)	$x_{kur}=\frac{\sum_{i=1}^{N}{\left(x_{i}-\bar{x}\right)}^{4}}{\left(N-1\right)x_{\sigma }^{4}}$
Minimum (S3)	$x_{\min}=\min\left|x_{i}\right|$	Skewness factor (S13)	$\alpha =\frac{x_{ske}}{x_{rms}^{3}}$
Mean value (S4)	$\bar{x}=\frac{1}{N}\sum_{i=1}^{N}x_{i}$	Kurtosis factor (S14)	$\beta =\frac{x_{kur}}{x_{rms}^{4}}$
Maximum (S5)	$x_{\max}=\max\left|x_{i}\right|$	Crest factor (S15)	$C_{f}=\frac{x_{p}}{x_{rms}}$
Root mean square (S6)	$x_{rms}=\sqrt{\frac{1}{N}\sum_{i=1}^{n}{x_{i}}^{2}}$	Shape factor (S16)	$S_{f}=\frac{x_{\mathrm{rms}}}{x_{\mathrm{av}}}$
Root amplitude (S7)	$x_{r}={\left(\frac{1}{N}\sum_{i=1}^{N}\sqrt{\left|x_{i}\right|}\right)}^{2}$	Impulse factor (S17)	$I_{f}=\frac{x_{p}}{x_{av}}$
Variance (S8)	$D_{x}=\frac{1}{N}\sum_{i=1}^{N}{\left(x_{i}-\bar{x}\right)}^{2}$	Clearance factor (S18)	$CL_{f}=\frac{x_{p}}{x_{r}}$
Standard deviation (S9)	$x_{\sigma }=\sqrt{\frac{1}{N}\sum_{i=1}^{N}{\left(x_{i}-\bar{x}\right)}^{2}}$	Coefficient of variation (S19)	$K_{v}=\sqrt{D_{x}}/x_{\mathrm{av}}$
Maximum to minimum difference (S10)	$x_{p-p}=\max x_{i}-\min x_{i}$		

Note: *x_i_* denotes the vibration signal sequence collected by the sensor, $x_{i}=[x_{1},x_{2},\ldots ,x_{N}]$. *N* denotes the number of data points.

**Table 2:** Frequency domain feature indexes used in this work.

Index	Function
Centroid frequency (S20)	$f_{c}=\frac{\sum_{k=0}^{N-1}f_{k}X\left(k\right)}{\sum_{k=0}^{N-1}X\left(k\right)}$
Average frequency (S21)	$f_{m}=\frac{1}{N}\sum_{k=0}^{N-1}X(k)$
Standard deviation of frequency (S22)	$\sigma_{f}=\sqrt{\frac{\sum_{k=0}^{N-1}{\left(f_{k}-f_{m}\right)}^{2}X(k)}{\sum_{k=0}^{N-1}X(k)}}$
Root mean square of frequency (S23)	$f_{rms}=\sqrt{\frac{\sum_{k=0}^{N-1}f_{k}^{2}}{N}}$
Variance of frequency (S24)	${\sigma_{f}}^{2}=\frac{\sum_{k=0}^{N-1}{\left(f_{k}-f_{m}\right)}^{2}X(k)}{\sum_{k=0}^{N-1}X(k)}$

**Table 3:** Calculated evaluation indexes using the eight sensitive feature indexes for the seven rolling bearings.

Bearing	Monotonicity Index	Correlation Index	Predictive Index	Robustness Index	Comprehensive Index	Original Maximum Comprehensive Index	Improvement
1-1	0.9458	0.9654	0.9428	0.8943	0.9482	0.8904	6.5%
1-2	0.8664	0.8628	0.9428	0.8805	0.8720	0.8173	6.7%
1-3	0.9593	0.9573	0.9428	0.8801	0.9490	0.8524	11.3%
1-4	0.8033	0.7707	0.9428	0.9097	0.8149	0.7518	8.4%
1-5	0.8779	0.8952	0.9428	0.8893	0.8924	0.8218	8.6%
1-6	0.9017	0.9132	0.9428	0.8982	0.9140	0.8096	12.9%
1-7	0.9167	0.8978	0.9428	0.8587	0.9059	0.8512	6.4%

**Table 4:** The details of the dataset used in this section selected from the IEEE PHM 2012 dataset.

Dataset 1 Load (N)	Rotation Speed (rpm)	Dataset 2 Load (N)	Rotation Speed (rpm)
4000	1800	4200	1650
Bearing	Actual life	Bearing	Actual life
Bearing 1-1	7 h 47 min 00 s	Bearing 2-1	2 h 31 min 40 s
Bearing 1-2	2 h 25 min 00 s	Bearing 2-2	2 h 12 min 40 s
Bearing 1-3	5 h 00 min 10 s	Bearing 2-3	3 h 20 min 10 s
Bearing 1-4	3 h 09 min 40 s	Bearing 2-4	1 h 41 min 50 s
Bearing 1-5	6 h 23 min 29 s	Bearing 2-5	5 h 33 min 30 s
Bearing 1-6	4 h 10 min 11 s	Bearing 2-6	1 h 35 min 10 s

**Table 5:** Remaining useful life prediction task description using IEEE PHM 2012 dataset.

Task	Training Bearing	Test Bearing
A	Bearing 1-1, 1-2, 1-3	Bearing 1-4
B	Bearing 1-1, 1-2, 1-3	Bearing 1-5
C	Bearing 1-1, 1-2, 1-3	Bearing 1-6
D	Bearing 2-1, 2-2, 2-3	Bearing 2-4
E	Bearing 2-1, 2-2, 2-3	Bearing 2-5
F	Bearing 2-1, 2-2, 2-3	Bearing 2-6

**Table 6:** Hyperparameter values of TCN–Transformer networks.

Hyperparameter	Value	Hyperparamete	Value
Batch Size	32	Epochs	10
Activation Function	GELU	Learning Rate	0.0001
Embedding Dimension	64	Hidden Unit Dimension	256
Temporal Window Length	30	Loss Function	MSE

**Table 7:** Comparison of results of TCN–Transformer and other advanced models.

Model	Average *RMSE*	Average *MAE*	Average *SCORE*
RNN	0.1093	0.0901	0.2129
LSTM	0.0969	0.0815	0.2004
GRU	0.0991	0.0831	0.2496
Dual-LSTM	0.1055	0.0728	0.2714
LSTM-AON	0.0873	0.0695	0.3286
BiGRU-GSA	0.0852	0.0634	0.4553
TCN-RSA	0.0765	0.0529	0.507
TFT	0.0602	0.0432	0.5614
TCN–Transformer	0.0514	0.0392	0.6346
IMP	14.62%	9.26%	13.04%

**Table 8:** Results of TCN–Transformer ablation experiment.

	*RMSE*			*MAE*			*SCORE*		
Task	TCN- Transformer	Transformer	TCN	TCN- Transformer	Transformer	TCN	TCN- Transformer	Transformer	TCN
A	0.0312	0.0177	0.6225	0.0227	0.0135	0.5131	0.6356	0.5290	0.1226
B	0.0660	0.0491	1.0182	0.0483	0.0406	0.8467	0.5673	0.5384	0.0724
C	0.0607	0.1208	0.9761	0.0530	0.1077	0.8184	0.5916	0.4122	0.0700
D	0.0275	0.1278	0.5127	0.0226	0.0624	0.4201	0.6764	0.5465	0.1961
E	0.0800	0.1257	1.0087	0.0436	0.0894	0.8567	0.6178	0.3828	0.0491
F	0.0430	0.0703	0.4298	0.0448	0.0662	0.3498	0.7188	0.5743	0.2675
Average	0.0514	0.0852	0.7613	0.0392	0.0633	0.6341	0.6346	0.4972	0.1296
*IMP*	39.67%			38.07%			26.63%		

## Data Availability

Data is contained within the article.
